# Hand Hygiene Habits of Ghanaian Youths in Accra

**DOI:** 10.3390/ijerph16111964

**Published:** 2019-06-03

**Authors:** Timothy B. Oppong, Haiyan Yang, Cecilia Amponsem-Boateng, Guangcai Duan

**Affiliations:** Department of Epidemiology and Biostatistics, College of Public Health, Zhengzhou University, Zhengzhou, Henan 450001, China; oppong.bonney@gmail.com (T.B.O.); amponsem-boatengc@aua.ac.ke (C.A.-B.); gcduan@zzu.edu.cn (G.D.)

**Keywords:** hand hygiene, hand hygiene habits, hand washing, hand sanitizer use

## Abstract

The human palm has been identified as one of the richest habitats for human microbial accommodation making hand hygiene essential to primary prevention of infection. Since the hand is in constant contact with fomites which have been proven to be mostly contaminated, building hand hygiene habits is essential for the prevention of infection. This research was conducted to assess the hand hygiene habits of Ghanaian youths in Accra. This study used a survey as a quantitative method of research. The findings of the study revealed that out of the 254 participants who fully answered the questionnaire, 22% had the habit of washing their hands after outings while only 51.6% had the habit of washing their hands after using the bathroom. However, about 60% of the participants said they sometimes ate with their hands while 28.9% had the habit of eating with the hand very often, a situation that put them at risk of infection from their hands since some participants had poor handwashing habits; prompting the need for continuous education on hand hygiene.

## 1. Background

The human hand has continual contact with the environment every day. It is also one part of the human body that is in constant communication with other parts of the body; including scratching the itching part, as well as serving the mouth with food and drugs. Research shows that the palm serves as the richest habitat for human microbial accommodation [1], making hand hygiene essential in infection prevention and control. Several pathogens such as *Staphylococcus* spp, *salmonella*, Escherichia coli, have been isolated from human palms; some of which were resistant to known antibiotics [2,3,4]. According to the Global Handwashing Partnership, forming hand hygiene habits means being able to undertake the procedures in hand hygiene automatically without involving the decision making parts of our brain [5]. This view conforms with the psychological understanding of habits formation and suggests that hand hygiene must become a habit, intrinsically performed by individuals whenever needed without it being as a result of decision making [6]. Hand hygiene habit for this research is defined as the regular and routine tendency to practice hand hygiene. Hand rub with alcohol-based hand sanitizers (ABHS) is also a recommended hand hygiene practice and considered the best option when hand washing is not possible; which is mostly the case when outdoors [7]. Fomites such as automated teller machines (ATM), computer keyboards, mobile phones generally serve as the source of infection in communities and the main source of nosocomial infections [2,8,9,10,11,12]. It is believed that most people do not wash their hands even when the activity they engage in warrants so [13]. Some studies attribute this to dirty and unavailable hand hygiene apparatus like water and washing basin [14,15]. Reports indicate that Ghana loses an estimated amount of 250 million US dollars due to factors like productivity loss and healthcare costs resulting from poor sanitation and diarrheal diseases [16]. Recently, there have been improvements in access to safe and clean water in Ghana; over 80% of the population now has access to safe drinking water [17,18], however, hand hygiene practices have not been maximized as cross-contamination from the hands has been reported in food handlers in Ghana [19,20]. The hand has also been identified to play a crucial role in the fecal-oral transfer of microbes among young children in Ghana [21] Hand hygiene practices in healthcare institutions have mostly been the focus of hand hygiene compliance campaigns since it is one of the major ways of dealing with nosocomial infections. Little attention, however, has been given to habitual hand hygiene compliance in the community especially in Ghana; a country where practices like open defecation and choked gutters are rampant; the prevalence of gastroenteritis and the cyclical outbreaks of cholera have been widely reported [16,22,23]. Therefore, this research was conducted to assess the hand hygiene habits of Ghanaian youths in Accra since a substantial portion of the Ghanaian population is considered to be youthful [24]; to determine if their hand hygiene habit puts them at risk of infection.

### 1.1. Research Questions

The study attempted to answer the following questions;

1. What are the hand hygiene habits of Ghanaian youths?
2. Are the hand hygiene habits of Ghanaian youths likely to put them at risk of infection from their hands?
3. What is the knowledge of Ghanaian youths about proper hand hygiene?
4. What recommendations can improve the hand hygiene habits of Ghanaian youths?

### 1.2. Objectives

1. To assess the hand hygiene habits of Ghanaian youths.
2. To determine if the hand hygiene habits of Ghanaian youths are likely to put them at risk of infection from their hands.
3. To assess the knowledge of Ghanaian youths on proper hand hygiene
4. Propose recommendations to improve hand hygiene habits of Ghanaian youths.

## 2. Materials and Methods

This study employed a survey method in cross-sectional research design.

### 2.1. Data Collection Process

Data collection was done through an online survey with the use of an online questionnaire. The researchers purchased an account with esurvaycreator.com after which a dedicated website was created for the purpose of the survey. The researchers created a semi-structured questionnaire on the site. A trial version of the site was used to test for validation on selected students social media groups before the account was purchased for the survey to begin. Changes were made to the questionnaire based on the responses and reactions of the trial respondents. The questionnaire (in Supplementary materials) contained 12 questions structured to assess the objectives of the study. Questions 1 and 2 gathered demographic information on gender and age group. Questions 3 and 4 assessed the hand hygiene habits of participants, questions 5 to 8 assessed if respondents were at risk of infecting themselves from their hands. Lastly, questions 9 to 12 assessed the knowledge of respondents on proper hand hygiene. The link to the website was then copied with an introductory message inviting people to take part in the survey. It was then broadcasted through several social media platforms including Whatsapp chat groups and Facebook wall posts, as they are the most common social media platforms to get most youths in Ghana [25]. Individual survey reports have revealed that about 65% of Ghanaians own mobile phones with nearly 45% connected to the internet through mobile phones alone [26]. Participants had a limited time of 2 weeks to fill out the questionnaire, of which they could start and continue at any time of their choosing within the two weeks. All the persons who participated in the stipulated two weeks period were included in the study. The researchers engaged the help of several group admins to post and repost the link at different intervals; between 6:30 a.m. to 8:30 a.m., 12:00 p.m. to 1:30 p.m. and then finally 5 p.m. until midnight for at least three times in a day for the two weeks period. Participants were promised absolute confidentiality.

### 2.2. Risk of Sampling Bias

The researchers employed several techniques in order to restrict the presence of several biases in the data collection. Firstly, the link was broadcasted on the reportedly most popular social media platforms in Ghana thereby making it accessible to most of the youths. Secondly, the link broadcast was done at times whereby social media users are active; that is, early morning when people are commuting to work, school, etc, lunchtime and in the evenings when people are returning home. This was done to restrict selection bias in the study sampling. Again, no rewards, tokens or payments were given to participants in order to reduce fake or rushed answers that normally result from participants who may answer research questions just for the token and thereby checking response bias. Lastly, the online platform also included several measures to prevent multiple participations from one individual participant.

### 2.3. Statistical Analysis

The data from the survey were extracted into an excel sheet for data cleaning. It was then extracted into SPSS where descriptive analysis like frequencies and averages were analyzed. Correlation analysis was also performed to identify the relationship between hand hygiene habits and other variables. Data analysis was done by using the Statistical Package for Social Sciences (SPSS) version 16.0 (SPSS Inc. 2007, Chicago, IL, USA).

## 3. Results

**Figure 1:**
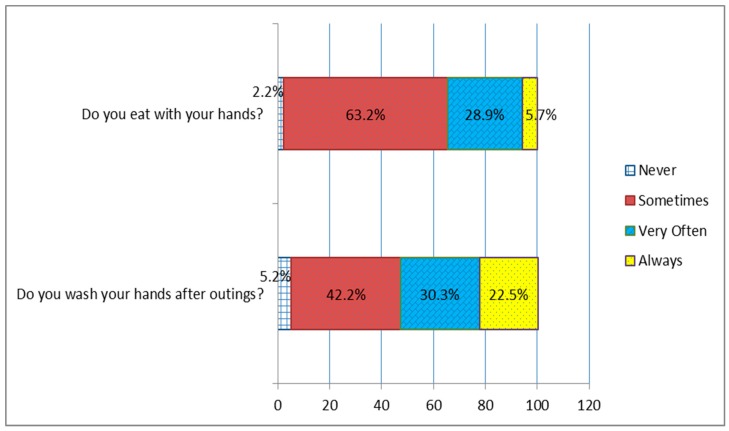
Diagram showing how often participants wash their hands after outings and how often they eat with their hands.

A total of 271 participants voluntarily participated in the survey but only 254 participants fully answered all questions in the survey hence statistical analysis focused on fully answered questionnaires. Of this figure, 143 (56.3%) were females while 111 (43.7%) were males. Majority 130 (51.2%) of the participants were aged between 15 to 25 years old followed by about 124 (48.8%) of the participants with the ages between 25 to 35 years. Over 61% of the respondents said they do not carry hand sanitizers with them; the majority (62.2%) of which were males. Only 98 (38.6%) of the respondents said they carried hand sanitizers every day with over 85% of them being females. There was a significant (0.470, *p* > 0.000) relationship between gender and the habit of carrying hand sanitizers (Table 1). Interestingly, less than 23% of the respondents said they always wash their hands after returning from an outing (school, work, church, etc.). There was a low correlation between gender, age and the habit of washing hands after an outing. However, a substantial portion of the participants always and very often preferred eating with their hands. Only six (2.4%) said they never eat with their hands. Figure 1 below shows details of how often participants wash their hands after outings and how often they prefer eating with their hands.

**Figure 2:**
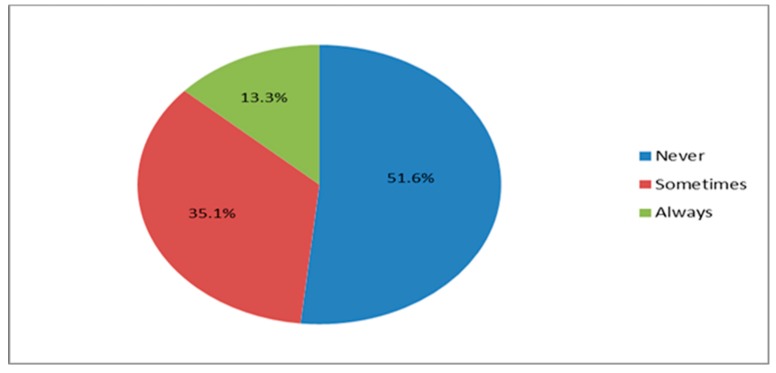
Frequencies of how often participants forget to wash their hands after using the toilet.

Almost all the participants 252 (99.2%) prefer eating with the right hand and about 87 (34.3%) of them said they wash only their right hand when they choose to eat with their hand while the remaining said they wash both hands before they eat with their hands. Interestingly, almost 50% of the participants either always or sometimes forgot to wash their hands after using the toilet. This habit moderately correlated with gender (Table 1). Figure 2 below gives details of their responses.

**Figure 3:**
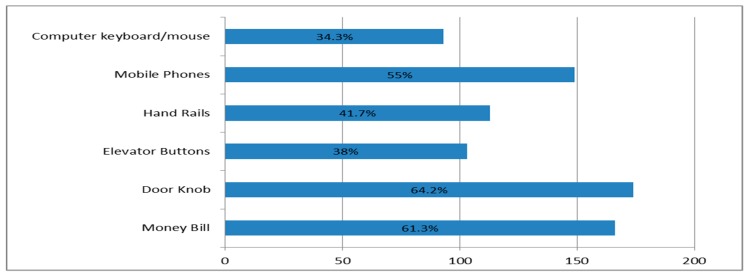
Participants views of sources of microbial contamination of the hands.

The majority of the respondents 208 (85.2%) agreed that it is best to wash the hands after both urination and defecation with the rest skewed towards just defecation. In general, most of the participants did not think that handrails, computer keyboard and mouse easily serve as sources of microbial contamination to the hand. However, the majority of them believed that money bills, door knobs, mobile phones and elevator buttons serve as an easy source of microbial contamination to the hand. Figure 3 below gives more details.

Again, the majority of the respondents 187 (76.6%) said they wash their hands under running water with soap. The remaining participants chose other methods which are not ideally recommended for proper hand washing. In addition, the majority of the respondents 188 (77%) said they had been taught how to wash their hands by a health professional.

## 4. Discussion

Hand hygiene is an essential part of the primary prevention of infections and to the best of our knowledge this is the first research that focused on assessing the hand hygiene habits in any group in Ghana. Though hand washing with soap remains the one most important action in controlling infections [27,28], the use of alcohol-based hand sanitizers (ABHS) has been reported to be effective in inactivating many types of microbes [29]. Even though ABHSs are not a replacement for hand washing with soap, especially in food settings [30], the right balance is to do a frequent hand rubbing with ABHS and wash the hands with soap under running water when the hand is visibly soiled or whenever possible [31]. Respondent’s hand washing habits could be characterized as low. Aside majority (61.4%) of our respondents not carrying ABHS on them, very few (22.5%) had a habit of washing their hands when they return home from an outing. A study comparing the microbial load in the palms of surgical doctors right before and after hand scrub before surgical procedures reported a considerable decrease in the microbial load in the palms of the doctors after hand scrub [32]. There was a positive association between gender and carrying of hand sanitizers. Results suggested that females are more likely to carry hand sanitizers than their male counterparts. This is consistent with a survey of college students that found an association between gender and handwashing behavior revealing that female students washed their hands more as compared to their male peers [33]. Hand hygiene education including ABHS use is also reported to have increased hand hygiene practice and especially ABHS use among participants who received education [34]. Again, nearly 36% and about 13% of the participants said they sometimes and always forget to wash their hands respectively, after using the bathroom. This is a poor hand hygiene habit since handwashing with soap after using the bathroom is a must for infection prevention [7]. Though the majority of the respondents showed good knowledge on the right way to wash the hands, there is still the need for hand hygiene education. This is to reinforce knowledge and build habits as knowledge does not imply compliance. This corroborates a study that found low levels of hand hygiene adherence within healthcare professionals in intensive care units [35]. Over 60% of the respondents did not consider computer keyboards, handrails and elevator buttons as a microbial source of infection however; there is research evidence that shows these fomites accommodate microbes [2,10].

### 4.1. Limitations of the Study

This study had several limitations such as the number of participants who decided to join the survey may not be directly representative of the population, calling for the need for a larger study. In addition, the data are presented with the assumption that participants answered truthfully without any way of practically corroborating their responses in the research, particularly in response to their knowledge of proper hand washing process. Lastly, the questionnaire used only underwent face validation; a kind of validation which is weak and does not check reliability and consistency.

### 4.2. Proposed Recommendations for Building Hand Hygiene Habit

*Objectives*

The objective of this recommendation is to build hand hygiene habit among the youths in the Ghanaian community. These points can also be applied in hospitals to develop hand hygiene habits in both patients and healthcare workers.

*Major Keynotes*

1. All public and private washrooms should have notices of reminders for users to wash their hands after using the facility. These notices should be placed in a very conspicuous place in the washroom so that users of the washroom cannot miss seeing it.
2. In addition, hand washing facilities in public places should be kept clean since some people have reported not washing their hands after using the toilet because the hand washing facilities were visibly dirty [15].
3. There should be a continuous hand hygiene education in communities to further enlighten more people on why and how to wash the hands.
4. Hand hygiene education should be focused on target groups to ensure maximum effect. Campaigners can target focus groups; for example gender-based groups like male-dominated jobs or environments (construction sites, mechanic shops, etc.) or female dominated environments like the market.
5. In addition, efforts should be made to generate hand hygiene habits among people in the community by ensuring repeated community engagements to serve as reinforcements.

## 5. Conclusions

Making hand hygiene a habit is an essential aspect of primary prevention of infections. Our findings showed that the majority of the respondents had not developed the habit of hand hygiene and this made most of them at risk of infecting themselves from their hands especially in a community where the majority of the participants sometimes prefer eating with their hands. It is therefore recommended that hand hygiene education will be intensified and also focused on helping the community to build hand hygiene habits.

## Figures and Tables

**Table 1:** Correlation between gender, age and hand hygiene habits.

(N = 254)	Do You Carry Hand Sanitizer on You Daily?			How often Do You Wash Your Hands after Returning from an Outing?					Do You Forget to Wash Your Hands after Using the Toilet?			
	Yes (n)	No (n)	Corr. Sig. (0.01)	Never (n)	Sometimes (n)	Very often	Always	Corr. Sig. (0.05)	Never (n)	Sometimes (n)	Always (n)	Corr.
**Gender**												
Male	14	97	0.470	12	51	35	13	0.135	40	49	22	0.281
Female	84	59	*p* > 0.000	2	53	42	46	*p* = 0.032	91	40	12	****
**Age**												
15–25	56	74	0.092	5	50	42	33	0.102	66	46	18	0.018
25–35	42	82	****	9	54	35	26	****	65	43	16	****

Note: Analysis without *p*-values was calculated with eta function in SPSS v. 16.0. Corr.: Correlation Coefficient. Sig: Significance level at. ****: *p*-value not applicable.

## Supplementary Materials

Supplementary materials can be found at https://www.mdpi.com/1660-4601/16/11/1964/s1.
