# Supplementary material for: Hand Hygiene Habits of Ghanaian Youths in Accra
Source: Int J Environ Res Public Health. 2019 Jun 3;16(11):1964. doi: 10.3390/ijerph16111964 (PMC6603880; doi:10.3390/ijerph16111964)
Supplement: Supplementary file 1 [file ijerph-16-01964-s001.pdf]

Hand Hygiene Habits of Ghanaian Youths in Accra

Page 1

Hello, thank you for taking the time to answer these questions to help with our survey. This 12 question survey has been put together to access the hand hygiene habits of Ghanaian youth in Accra. Hand hygiene has been identified as the most important single action one can take to prevent the spread of many infections like cholera and many other diarrheal diseases which burdens the Ghana Health Service. Kindly take some few minutes to answer these questions to aid us in this survey. Please answer these questions as sincere as you can. Be assured that your identity is totally protected. Thank you very much for your time.

Page 2

1. What is your Gender? *

Male

Female

2. How old are you? *

15 - 25

25 – 35

Above 35*** (Participants above 35 are redirected to last page of survey)

3. Do you normally carry a hand sanitizer with you? *

yes

no

4. How often do you wash your hands when you return from school, work, church, etc? *

Never

Sometimes

Very Often

Always

5. How often do you eat with your hands? *

Never

Often

Very often

Always

6. When you eat with your hand, which hand do you use? *

Right hand

Left hand

7. Which of your hands do you wash before eating? *

Choose the one you do mostly

Right Hand

Left Hand

Both Hands

Page 3

8. Do you often forget to wash your hands after using the bathroom? *

Never

Sometimes

Always

9. Which of these activities require proper hand hygiene afterwards? *

Kindly write your answer in the box below

Defecation

Urination

Both

10. Which of these objects do you think easily serve as sources of microbial contamination your hand? *

Choose all that apply

Money bill

Door knob

elevator buttons

Hand Rails

Mobile Phones

Computer keyboard and mouse

11. How do you wash your hands? *

In a bowl of water with soap

In a bowl of water without soap

Under a tap or running water without soap

Under a tap or running water with soap

Other

12. Have you ever been taught by a professional how to effectively wash your hands? *

yes

no

You have completed the survey. Thank you very much for your participation.

You can now close the window.
